# Abdominopelvic Splenosis—An Unusual Cause of Tenesmus

**DOI:** 10.1007/s11605-014-2548-7

**Published:** 2014-05-29

**Authors:** King Kenneth Cheung, Thomas Wagner, Margaret Hall, Lee Dvorkin

**Affiliations:** 1 Department of Nuclear Medicine, Royal Free Hospital, London, UK; 2 Department of Surgery, North Middlesex Hospital, London, UK; 3 Centre for Medical Imaging, University College London, 3rd Floor East, 250 Euston Road, London, NW1 2BU UK

**Keywords:** Splenosis, Colloid, Tenesmus, Splenunculi

## Abstract

Splenosis is a rare condition defined as seeding and autotransplantation of splenic tissue, typically after blunt abdominal trauma (e.g. from road traffic collision). Sites of splenosis ranging from intrathoracic to intrapelvic have been reported, and symptoms vary greatly depending on the site and size of lesions. We present the use of Tc-99m sulphur colloid SPECT/CT in diagnosing a case of multiple abdominopelvic splenosis as the cause of new-onset tenesmus and constipation, which was initially thought to be due to colorectal malignancy, 47 years following the initial abdominal trauma.

A 63-year-old man presented with new-onset tenesmus and change of bowel habit with increasing constipation. He has no significant past medical or surgical history apart from hospitalisation as a teenager for a road traffic accident. Clinical examination including a rectal examination revealed a smooth, soft mass at the anterior rectum with no blood or melena. Blood tests including CBC and CEA were all within normal limits. Colonoscopy showed a focal protrusion of bowel wall with normal mucosal appearance within the anterior rectum, which corresponds to the soft tissue mass palpated during rectal examination. A contrast-enhanced CT revealed multiple well-defined soft tissue lesions within the abdomen and pelvis, resembling malignant mesenteric nodes or deposits. Given the patient’s history of post-traumatic splenectomy, splenosis was suspected. Tc-99m sulphur colloid SPECT/CT was performed and demonstrated multiple foci of uptake within the soft tissue nodules described on CT in the left paracolic gutter, the anterior abdomen and pelvis predominantly (Fig. 1a–d). In addition, two further foci were found abutting the rectum (Fig. 1e, f). The findings were consistent with disseminated abdominopelvic splenosis (Fig. 2).

**Fig. 1:**
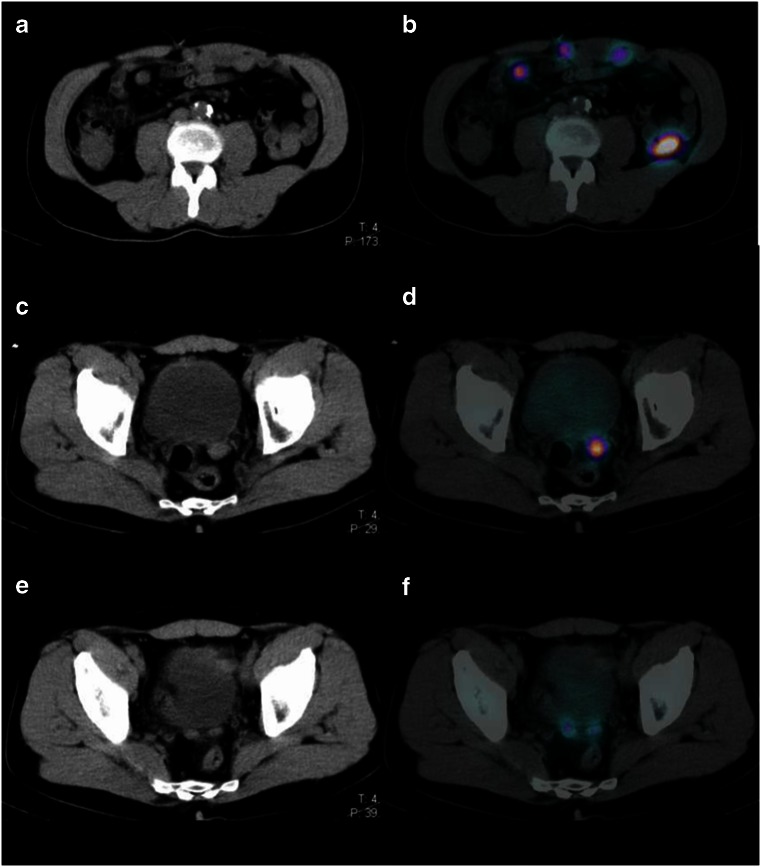
**a**–**f** CT of the abdomen and pelvis with Tc99m colloid SPECT overlay, demonstrating avid uptake of tracer

**Fig. 2:**
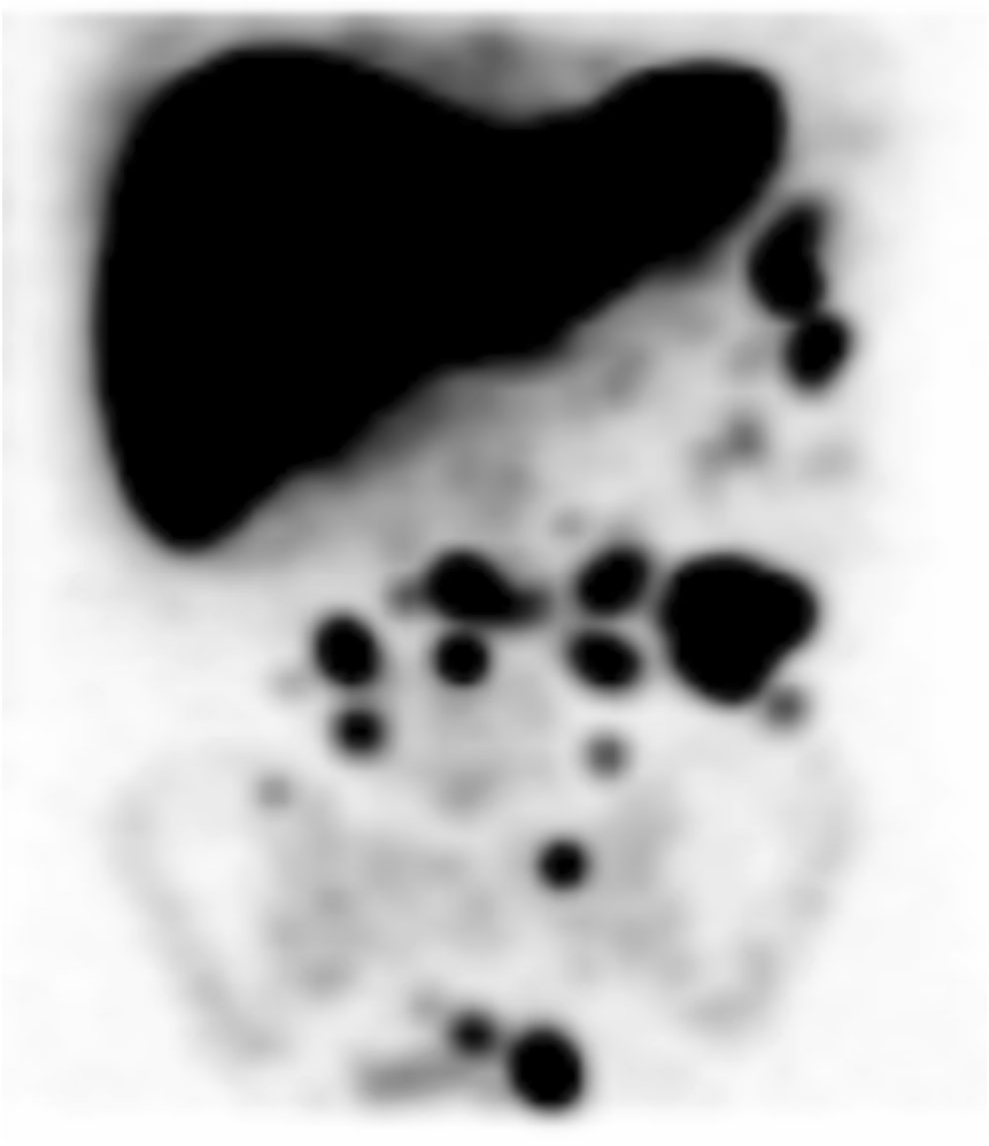
Maximum intensity projection of SPECT giving an overview of the extent of disseminated splenosis within the abdomen and pelvis

Splenosis is a rare condition where seeding and autotransplantation of splenic tissue occurs typically after blunt abdominal trauma. Tc-99m sulphur colloid and heat-denatured erythrocytes (dRBC) scintigraphy are two highly accurate and non-invasive methods for evaluating splenosis based on sequestration and phagocytosis of these agents in the reticuloendothelial system,^1^^,^ 2 with the latter allowing differentiation of hepatic tissue from splenic tissue.^3^^,^ 4 The use of SPECT/CT greatly improves visualisation and localisation of tracer uptake, thus yielding higher diagnostic accuracy.^5^ Although a rare condition, splenosis should be suspected when a radiological finding of well-defined intra-abdominopelvic nodules is accompanied with a history of splenic injury. Here, a diagnosis was reached non-invasively by combining careful history taking and highly specific scintigraphy, and invasive interventions were avoided. The patient was managed conservatively.

## References

[1] Armas RR (1985). Clinical studies with spleen-specific radiolabeled agents. Semin Nucl Med..

[2] Gunes I, Yilmazlar T, Sarikaya I (1994). Scintigraphic detection of splenosis: superiority of tomographic selective spleen scintigraphy. Clin Radiol..

[3] Hagman TF, Winer-Muram HT, Meyer CA (2001). Intrathoracic splenosis: superiority of technetium Tc 99 m heat-damaged RBC imaging. Chest..

[4] Ksiądzyna D (2011). A case report of abdominal splenosis—a practical mini-review for a gastroenterologist. J Gastrointestin Liver Dis..

[5] Horger M, Eschmann SM, Lengerke C (2003). Improved detection of splenosis in patients with haematological disorders: the role of combined transmission-emission tomography. Eur J Nucl Med Mol Imaging..

